# Effect of copper rod length on the melting behavior of paraffin wax in hemispherical latent heat storage units

**DOI:** 10.1038/s41598-026-43858-1

**Published:** 2026-03-17

**Authors:** Abbas Fadhil Khalaf, Farhan Lafta Rashid, Mushtaq K. Abdalrahem, Hayder I. Mohammed, Ephraim Bonah Agyekum, Atef Chibani, Ahcene Keziz, Ali Ismael, Taha Abdel Mohaymen Taha

**Affiliations:** 1 Department of Petroleum Engineering, College of Engineering, University of Kerbala, Karbala, 56001 Iraq; 2 Department of Statistics, College of Administration and Economics, University of Kerbala , Karbala, Iraq; 3 College of Pharmacy, University of Al-Ameed, Karbala, Iraq; 4 Physics Department, College of Education, University of Garmian, Kurdistan Region, Kalar, 46021 Iraq; 5 Istanbul Okan University Tuzla Campus, 34959 Istanbul, Turkey; 6 Western Caspian University, 31, Istiglaliyyat Street, AZ1001 Baku, Azerbaijan; 7 Research Center Industrial Technologies CRTI, Cheraga, P.O. Box 64, 16014 Algiers, Algeria; 8 Department of Physics, Physics and Chemistry of Materials Laboratory, University Pole, 28000 M’sila, Algeria; 9 Physics Department, Lancaster University, Lancaster, LA1 4YB UK; 10 Physics and Engineering Mathematics Department, Faculty of Electronic Engineering, Menoufia University, Menouf, 32952 Egypt

**Keywords:** Phase change material (PCM), Thermal conductivity, Numerical simulation, Ansys fluent, Thermal augmentation, Energy storage efficiency, Energy science and technology, Engineering, Materials science, Physics

## Abstract

The inherently low thermal conductivity of phase change materials (PCMs) stills a major limitation for their use in latent heat thermal energy storage (LHTES) units, as it significantly reduces the charging procedure and decreases system responsiveness. This study presents a novel numerical examination of the isolated effect of a single vertical copper rod of varying lengths on the melting performance of RT42 paraffin wax within a hemispherical cell. Unlike previous studies that focused on fins or composite structures in other geometries, this work uniquely quantifies the impact of systematically increasing rod length (0, 10, 20, and 30 mm) on melting enhancement in hemispherical configurations. Four cases had been simulated using ANSYS Fluent 16 with enthalpy-porosity technique to obtain transient heat transfer, fluid flow, and evolution of melting front. It was shown that the copper rod boosted thermal exchange and shortened the total time of melting, and it took 300 min (no rod) to 150, 120, and 90 min when using 10, 20, and 30 mm copper rod, respectively. The highest reduction in melting time was 70 percent and the corresponding increment in the melting rate was 233 percent in the longest rod compared to the finless. Besides, the existence of the rod resulted in the existence of more homogeneous temperature distributions and stronger convective currents, which increased the overall thermal efficiency. The findings provide valuable design guidance, demonstrating that the thermal conductivity limitation of organic PCMs can be effectively overcome through simple geometric modifications using high-conductivity inserts.

## Introduction

The recent demand of energy-saving thermal management has led to the intensive study of phase change materials (PCMs) to be used as latent heat thermal energy storage (LHTES)^1,2^. The phase transition properties of PCMs provide special benefits in temperature regulation, and are used in the building envelope to control indoor climate and battery thermal management systems in which a controlled heat uptake and release is essential to system safety and performance. Nevertheless, their overall application is limited due to their low thermal conductivity, which is usually lower than 1 W/m.K. thermal conductivity; this is critical for the entire LHTES system due to its low level of responsiveness because it greatly hinders the movement of heat during melting and solidification operations^3,4^. In order to overcome this weakness, scholars have studied some of the techniques of improving these designs, and one of the best improvement methods has been the addition of metallic fins. The metal fins are conductive channels that transport heat to deeper within the PCM volume and suppress the effects of slow natural convection leading to significantly increased melting rates and uniformity^5,6^. One material that has enjoyed extensive use in the design of fins is copper, which has a thermal conductivity of about 400 W/mK, and copper foam fins have proven to be particularly effective in speeding up thermal transfer due to its combination of high conductivity and large surface area^7,8^. In addition to choice of material, the geometric properties of fins (size, length and thickness) have a profound influence on overall system performance, and optimization of these design designs allows the attainment of maximum thermal gain and minimum added weight and material cost.

Particularly the composite metal fins, in the encapsulations of PCM on a small scale, used to store energy, have been studied recently, and experimental and numerical studies indicate that well-designed fins adapted to the geometries of a sphere can dramatically raise the melting power output^9–11^. Analytical studies have played a significant role in the explanation of the underlying mechanisms, which verify that the geometry and structure of the fins have significant roles in dictating thermal exchange rates^12^. In addition to the normal straight fins, scientists have investigated angled and non uniform distributions of fins to overcome non-uniformity in heating, and the experimental evidence shows that angled fins enhance the spread of thermal energy and enhance a full phase change^13^. Likewise, pin–fin designs in spherical cold storage application capsules have demonstrated several times greater thermal performance, and fin length, diameter and location are directly proportional to heat transfer-rates and heat storage capacity^14^. These design lessons are useful pointers to engineers who need to optimize thermal storage of PCM-based systems.

Similar developments in related energy disciplines have supported the significance of geometric optimization rules. As an example, the algae pyrolysis research in the alkaline molten salt reactors has already shown innovative methods of controlling heat that can be applied not only to the conventional PCM systems but also theoretical and numerical simulations of printed circuit heat exchangers have offered the background knowledge on fin effectiveness in straight-channel setups^15^ and^16^. Fin-tube designs have been compared across different designs and thermal conductivity of copper has always been a critical performance differentiator^17^. These multidisciplinary observations make PCM optimization lie in a wider spectrum of heat transfer studies in which the choice of materials and structure design meet to facilitate high-performance systems.

The concept of geometric innovation has gained more significance in the development of spherical PCM capsule, and channel design optimization studies have developed definite correlation between geometric parameters and thermal performance at a balance between efficiency and manufacturing cost^18^. An interesting development has been hollow fin capsules, which introduce multiple conductive channels, but at a low material cost relative to solid fins, and which have been shown by numerical simulation to allow the exploitation of both conduction and convection modes of operation to achieve good performance efficiency tradeoffs^19^. Other methods are dynamic rotation of PCM storage systems, where experimental results in winter seasons showed better thermal performance in terms of improved convection patterns^20^, timing studies have shown that optimized rotation cycles in coordination with charging and discharging processes have a major impact on system responsiveness^21^. These are the examples of how mechanical interventions can be compatible with geometric innovations to attain better PCM performance.

The PCM design strategies are greatly progressed through both theoretical and numerical modeling. Heat transfer enhancement of the fins has extensive literature that can be used to predictively optimize geometry and placement to achieve the required performance^22^. Asymmetric fin designs have been showing strategic benefits in the local heat exchange, addressing the inefficiencies at uniform layouts^23^. Geometric and material engineering designs that incorporate stair-like fins with nanoparticles, as demonstrated by numerical experiments, are becoming increasingly popular, and they show significant positive changes in the PCM charging rates and storage capacity^24^. The practical guidelines have been determined to be experimental confirmation of fin disposition in the sphere of the capsules to develop an effective arrangement of the fins in terms of manufacturing and space limitations^25^. Nanoparticles in PCM systems have been extensively studied to understand the interactions of complex heat transfer in packed bed energy storage systems^26^. Computational fluid dynamics (CFD) has become a fundamental instrument in the assessment of new fin shapes to allow the detailed study of thermal exchange and structures of flows in the process of charging PCM^27^. Developed designs are still on the way such as the use of double-layered PCM capsules with an annular fin that has shown to have a higher storage capacity and efficiency and has been designed to approach future directions in PCM system development^28^.

The study of Belazreg et al.^29^ compared the performance of different complex fin shapes adopted in the PCM unit enhanced PV panel with that of a simple plate fin. The fin configurations examined are basic plate fins (BC), a tree-fin with two separate branches at 45 (C1), a tree-fin with two separate branches at 90 (C2) and aligned with the middle of the thermal unit and a tree-fin with two separate branches at 90 (C3) and y with rounded corners and longer than the middle of the thermal unit. The results showed the addition of the proposed fins containing PCM increased the melting rate of PCM and elevated the PV efficiency. Out of the four fin configurations examined it was clear that the C1 fin configuration performed the best amongst the other fin configurations.

Qasem et al.^30^ examined how T-fin concentration affected the melting behavior of the nano-enhanced phase-change material (NePCM) composites. The heat flux on a rectangular storage space was tested on with nano additions of Cu. Different parameters were considered including fin levels, concentration of Cu, tilt angle and intensity of heat flux. To determine the effect of the use of T-fins on the heat transfer mechanism that governs the melting of PCM, the study involved six performance metrics, which are the liquid fraction, temperature, velocity, energy stored, Bejan number, and average Nusselt number. The enthalpy-porosity model was used to simulate the phase change process. The results show that the three-level T-fins has a speed of melting that is by 5% higher and an energy storage capacity 42.6 percent greater compared to one-level T-fins.

Qasem et al.^31^ evaluated numerically the performance of a vertical latent heat thermal energy storage (LHTES) system with twisted inner tubes arranged in a two-tube system. The five twist angles (0, 360, 720, 1080 and 1440 on 25 cm tube length) were studied to evaluate the effect on phase changes, thermal distributions, and storage ability of thermal energy using n-octadecane as the phase change material (PCM) and water as the heat transfer fluid. The results showed that the twist system greatly promoted convective heat transfer mechanism in the LHTES, which speeded the phase change and thermal activity. The twist structure of 1080, i.e., it had better performance with 30.1 percent decreasing solidification time, 16.9 percent decreasing melting time and 8.5 percent decreasing energy discharge time relative to the base straight tube.

Although several studies have explored the complex fin geometry or composite structures in a cylindrical or rectangular enclosure; no research has examined the length of one simple vertical copper rod in isolation and in a quantitative manner on the melting behavior within a hemispherical latent heat storage unit. This paper offers the first numerical data demonstrating that such a simple geometrical parameter as rod length can be optimized to achieve significant performance improvements (70 percent lower melting time, 233 percent faster melting rate), and is a cost-effective, low-cost design strategy of compact LHTES systems, not previously measured quantitatively.

Although the overall advantage of fins and high-conductivity inserts in different geometries was proved previously, the independent and systematic influence of the length of a single internal rod, being the only variable, on the melting dynamics of a hemispherical enclosure, was not studied. The rod length is important since it directly regulates the depth of conductive penetration into the PCM core, thus regulating the transition between conduction and convection dominated melting, a process the sensitivity of which to this single geometrical parameter has not been measured. The open physical phenomenon is the exact correlation between the small increases in rod length and the associated increase in coupled conduction–convection heat transfer, which this research has uniquely measured in order to define a clear design criterion.

## Numerical technique

### Physics models

**Fig. 1:**
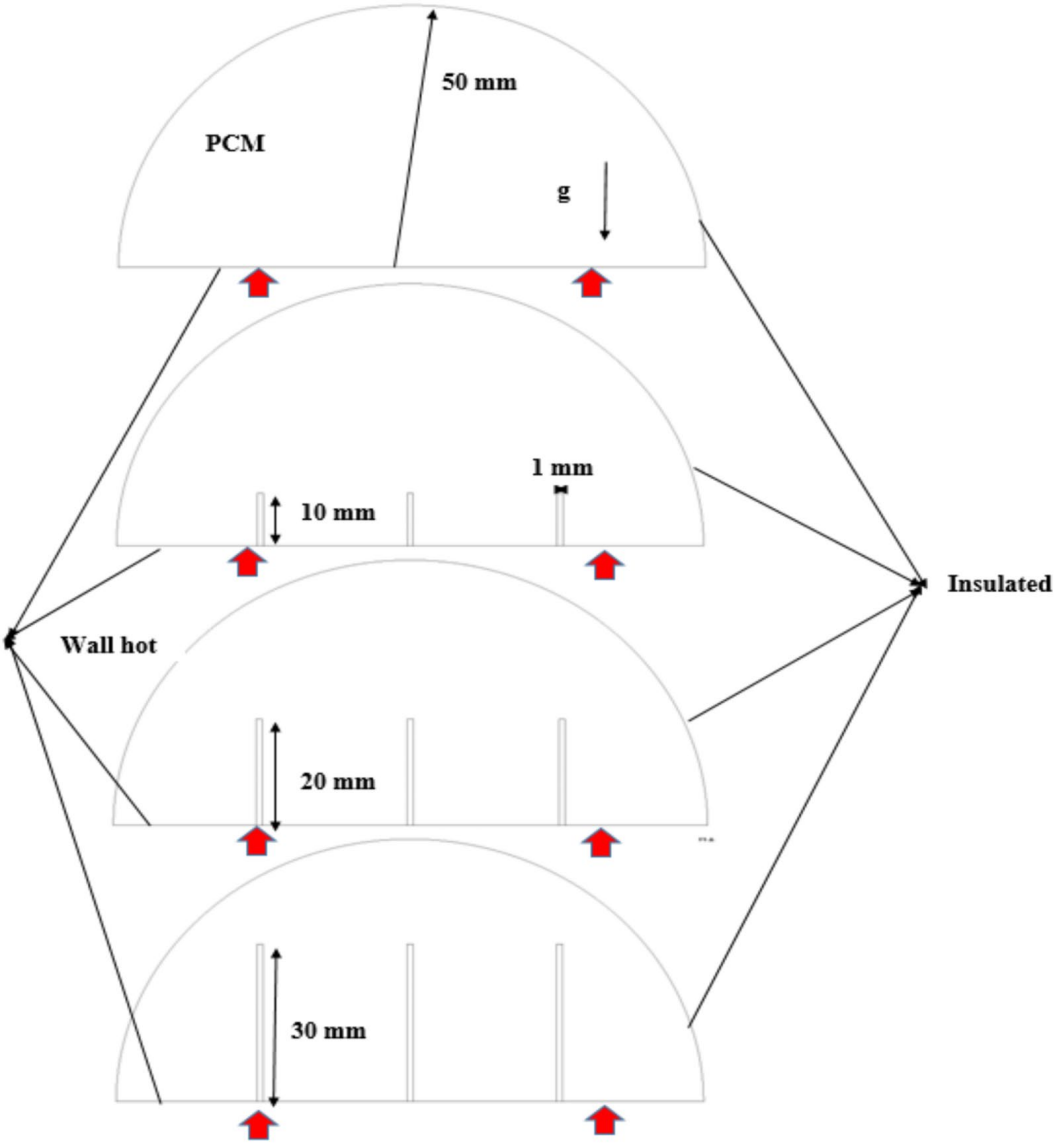
Configuration of physical model.

The physical model under consideration consists of a vertically placed hemispherical cell with a radius of 50 mm, which is occupied with the selected PCM, RT42 paraffin wax. To investigate the effect of conductive enhancement, a centrally positioned copper rod is introduced into the PCM domain, and its length is scientifically diverse to analyze its impact on the charging process. Specifically, three rod lengths (10, 20, and 30 mm) are considered, in addition to a baseline case without a rod for comparison. The copper rod, with a diameter of 1 mm, serves as an internal fin that extends vertically from the heated flat wall of the hemispherical cell, conducting thermal energy directly into the PCM core. The flat boundary at the bottom of the hemisphere is exposed to a uniform temperature source, representing the hot wall. In contrast, the curved periphery of the hemisphere is thermally insulated to ensure unidirectional thermal exchange from the heated surface toward the interior. This setup enables a controlled investigation of the role of rod length in modifying thermal gradients, free convection designs, and overall melting dynamics within the PCM. Figure 1 illustrates the geometry of the model and the spatial placement of the copper rod, highlighting the variations in rod length, the heating boundary, and the insulated surfaces that form the boundary conditions of the system.

### Computational technique

The capacity to prediction details of the charging processes that performed in the hemispherical cell comes from the numerical study. It was revealed that the flow was laminar, unstable, incompressible, and two-dimensional. To perform charging processes, it is presumed that the liquid and solid phases are homogeneous, isotropic, and maintain thermal equilibrium at their interface. The phase-change zone of the PCM was selected to track the enthalpy-porosity path. The solid–liquid interface’s constant mobility, nonlinearity, and temporal conduct are all characteristics of the PCM charging methods, which are considered a congregation of original challenges. The melting processes of PCMs are modelled by considering the simultaneous continuity, momentum, and energy governing partial differential equations, which are formulas^32–35^.

1 $$\frac{\partial \rho }{\partial t}+ \nabla . \left(\rho V\right)=0$$

2 $$\frac{\partial (\rho v)}{\partial \mathrm{t}}+ \nabla . \left(\rho V\right)= - \nabla P+ \mu {\nabla }^{2}V+ \rho g+S$$

3 $$\frac{\partial }{\partial t}\left(\rho H\right)+ \nabla .\left(\rho VH\right)= \nabla .\left(K\nabla T\right)$$

The specific enthalpy *H* is the sum of the sensible enthalpy (h) and the latent heat (Δ*H*),

4 $$H\,\, = h\,\, + \,\Delta H$$

where,

5 $$h={h}_{ref}+\underset{{T}_{ref}}{\overset{T}{\int }}{C}_{p} dT$$

6 $$\Delta {\text{H = }}\beta {\mathrm{L}}_{{\mathrm{f}}}$$

7 $$\beta = \left\{ \begin{gathered} 0\,\,\,solidus\,if\,\,T < \,T_{s} \hfill \\ 1\,\,\,liquidus\,if\,\,T\, > T_{l} \hfill \\ \frac{{T - T_{S} }}{{T_{l} - T_{s} }}\,\,if\,\,T_{s} \le \,T\, \le {\rm T}_{l} \hfill \\ \end{gathered} \right.$$

the liquid fraction (*β*) can be written as^36–38^.

The term S in Eq. 2 is the Darcy’s law damping part that is added to the momentum formula because of the phase change impact on the convection. Introduced by^39–41^.

8 $$S=\frac{{C(1-\beta )}^{2}}{{\beta }^{3}}V$$

where, the coefficient (C) is a mushy zone constant (10^4^ to 10^7^), which shows the geomorphology of the charging forepart. In this work C is set to 10^5^.

### Boundary conditions

The hemispherical cell that was deliberate is insulated on all sites except one which the heat passes to the phase change materials. The temperature (47 °C) is passed on the wall. A paraffin wax (RT42) is used as a phase change material. The thermal properties of paraffin are listed in Table 1.

**Table 1:** Thermal properties of the Paraffin (RT42)^42^.

Properties	RT42
Density, *ρ* (kg/m^3^)	760
Specific heat capacity, *C*_*p*_ (J/kg.K)	2000
Thermal conductivity, *k* (W/m.K)	0.2
Dynamic viscosity, *μ* (kg/m.s)	0.02351
Thermal expansion rate, *α* (1/K)	0.0005
Latent heat,* L* (J/kg)	1,65,000
Melting temperature, *T*_*m*_ (K)	311.15 – 315.15

### Assumptions

**Fig. 2:**
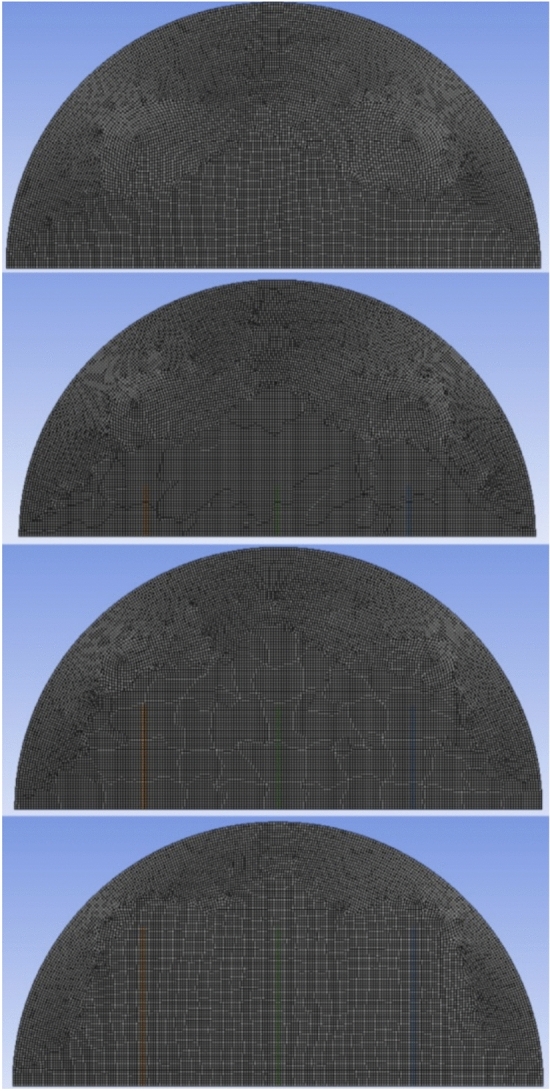
Formation of mesh pattern.

The assumptions that are considered in rising a mathematical model that describes the charging procedure within a hemispherical cell include: flow is unsteady, laminar, and incompressible; the viscous dissipation is insignificant; any change in volume by the transition of solids into liquids is ignored; the environment does not lose or gain heat; and the thermal characteristics of the phase change material are held constant in both solid and liquid phases- Fig. 2 represents the mesh supply of the examined model.

In the current work, the laminar flow assumption is explained by the estimated Rayleigh number (Ra) of the molten PCM layer, which was determined to be of the order of 10^5^–10^6^, and does not exceed the standard transition number to turbulence (10^9^) in natural convection in enclosures.

### Grid independence and the code validation tests

**Fig. 3:**
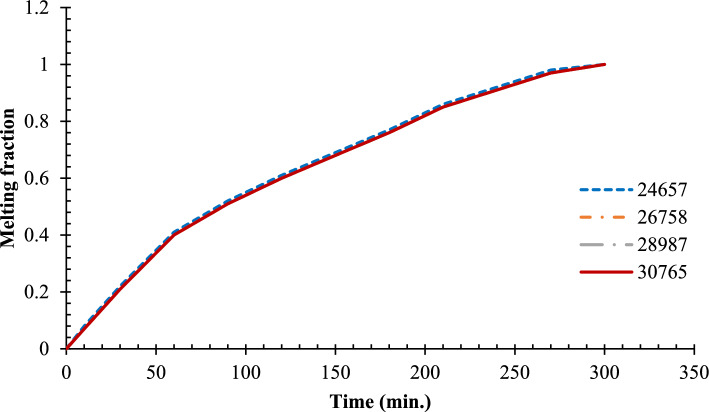
Grid independence without fins.

A mesh independence test was carried out to validate and verify the trademark of the CFD simulations by testing the four mesh densities using 24,657, 26,758, 28,987, and 30,765 elements. This was aimed at evaluating the effects of mesh resolution on the phase change behavior inside the hemispherical cell. The results showed that the development of the phase change was similar across all mesh densities, indicating that the solution was not dependent on the mesh size. Therefore, the mesh of the minimum number of elements (26,758) was used in all the subsequent computations to save on computer time and still achieve quality results, as shown in Fig. 3. It is noteworthy that grid independence test aims at confirmation of numerical convergence and not an established percentage improvement in the number of elements. The chosen mesh progression is a systematical and monotonic refinement, at the same time, high quality of elements is preserved, especially at the areas with high thermal and velocity gradient around the copper rod and solid/liquid boundary. The similarity in the liquid-fraction histories of all meshes used in the experiment shows that additional refinement does not change the project predicted melting behavior, which means that discretization errors are insignificant. Hence, the 26,758 element mesh was chosen as a good balance between precision and computational efficiency of the number.

The GCI values of the liquid fraction at the representative times steps using the three best meshes (26,758, 28,987 and 30,765 elements) were obtained as less than 3 percent, which confirms that the solutions are in the asymptotic range of convergence. We also have measured L2 error of the temperature field between successive mesh refinements, which was found to decrease monotonically with mesh refinement and was less than 2.5 percent of the initial error in the desired mesh of 26,758 elements. These numerical measurements confirm that the chosen mesh gives a grid-independent solution having a discretization error inside the acceptable range, which proves that our numerical forecasting is valid.

This part is aimed at the validation of the numerical findings that were provided on ANSYS/FLUENT 16 simulations against the available experimental outcomes and theoretical estimates that can be related to the melting of PCMs. A quantitative comparison of the temporal evolution of the liquid fraction between the present numerical predictions and the experimental measurements of Dhaidan and Khalaf^32^ was performed, yielding a maximum relative error of less than 5.2% and an average absolute deviation of 3.8% over the melting period. This close agreement confirms the accuracy and reliability of the present enthalpy-porosity model in capturing the phase change behavior within the hemispherical geometry.

## Results and discussion

**Fig. 4:**
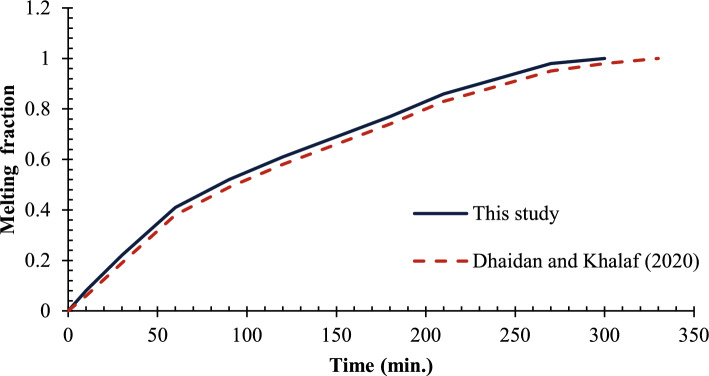
Comparison of the developing the liquid fraction for this study against the research^32^.

Four different scenarios were considered in this work, where the hemispherical cell was filled with phase-change materials, first we will analyze the cell without copper rod to have a reference point and then the three other cases would involve adding copper rods of different lengths in the cell to determine their effects on the charging process and total time requested to melt a cell Fig. 4.

### Case one (the cell without rods)

**Fig. 5:**
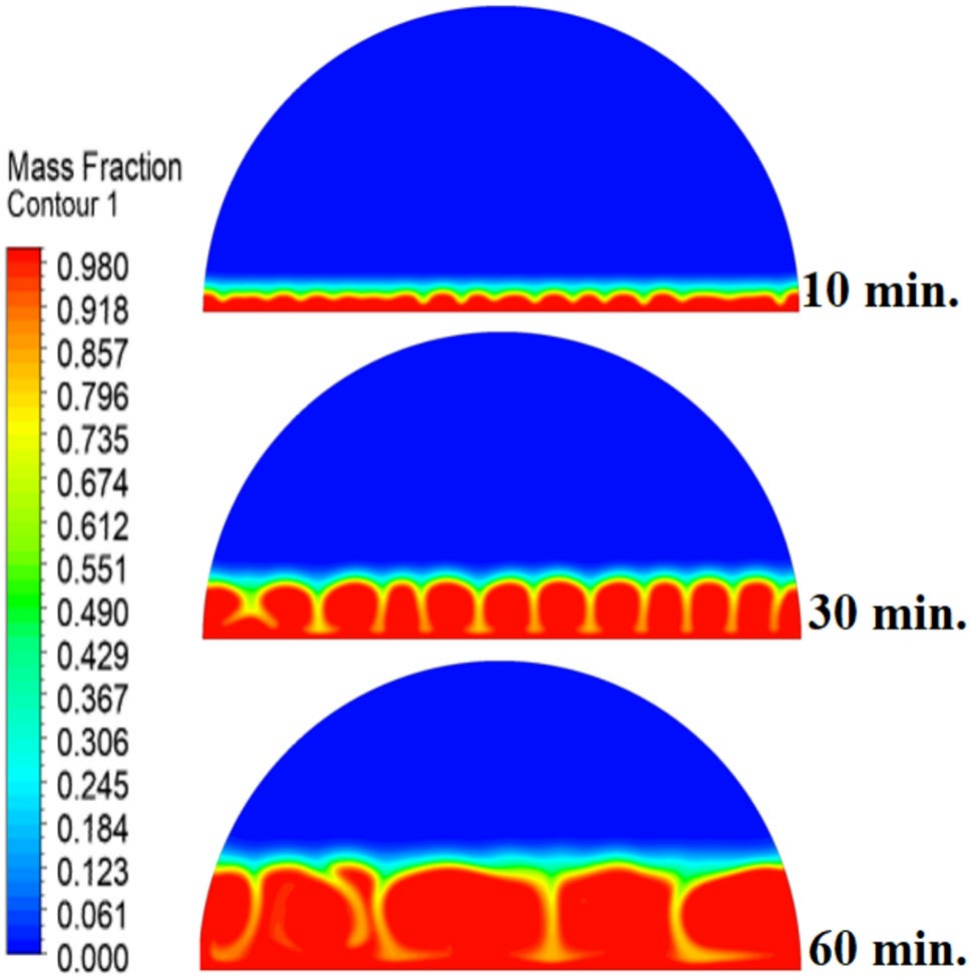
Predicted evolution of the melting process without rods.

Figure 5 demonstrates how the expected charging process would change in the absence of a copper rod, whereby the process would start melting close to the heated wall by means of conduction, followed by inward progression mainly by natural convection. It is also quite slow, with the total time to complete melting amounting to 300 min, owing to the decreasing efficiency of heat transfer at a distance from the wall because of the dependence on buoyancy-induced flows and the weak conductivity behaviour of the PCM itself.

**Fig. 6:**
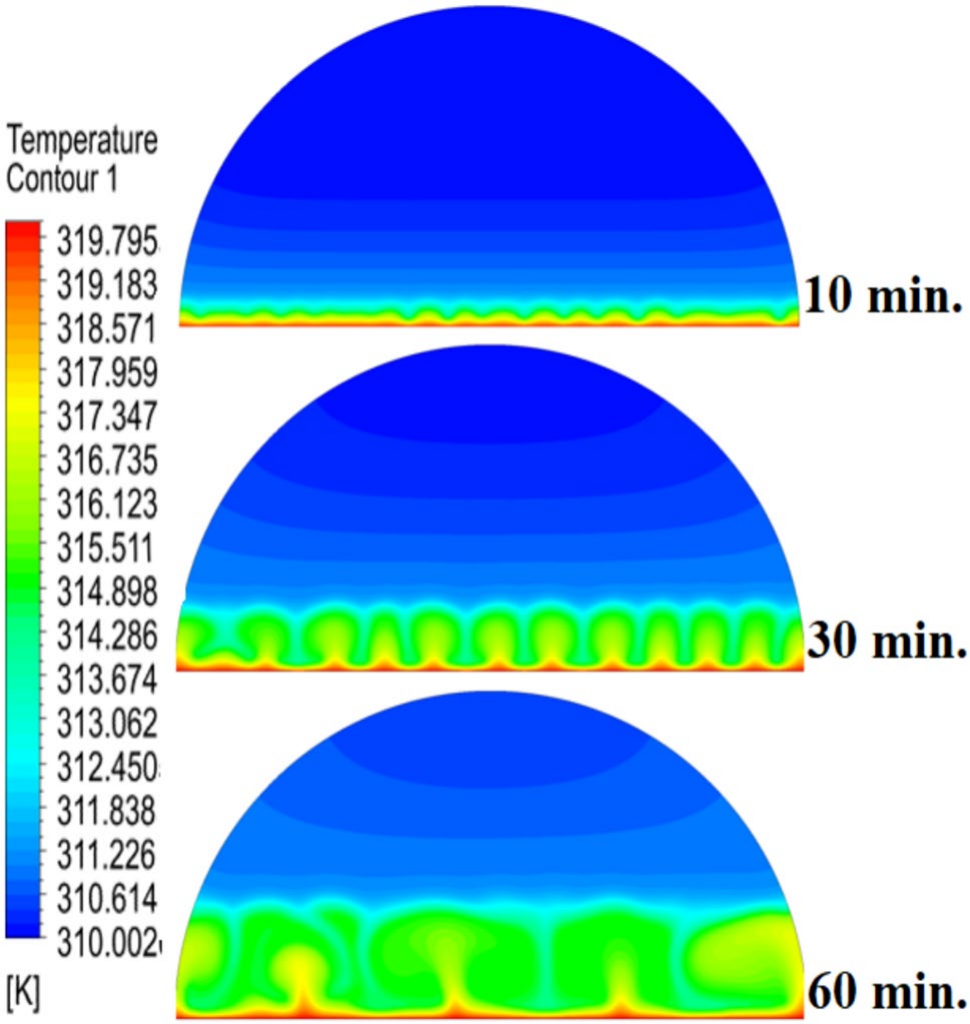
Temperature distributions with and without rods.

Figure 6 illustrates the temperature outline of the hemispherical cell without a copper rod, and the thermal gradient is steep from the warm wall (held at 47 °C) to the inner parts. This trend is because of the low conductivity of paraffin wax (RT42, k = 0.2 W/m.K), which causes heat transfer to be dependent on natural convection, an inefficient process. This means that temperatures drop very fast beyond the wall, with the PCM being a substantial insulator that slows the spread of heat into the heart of the cell, leaving a vast core of the cell thermally stagnant.

**Fig. 7:**
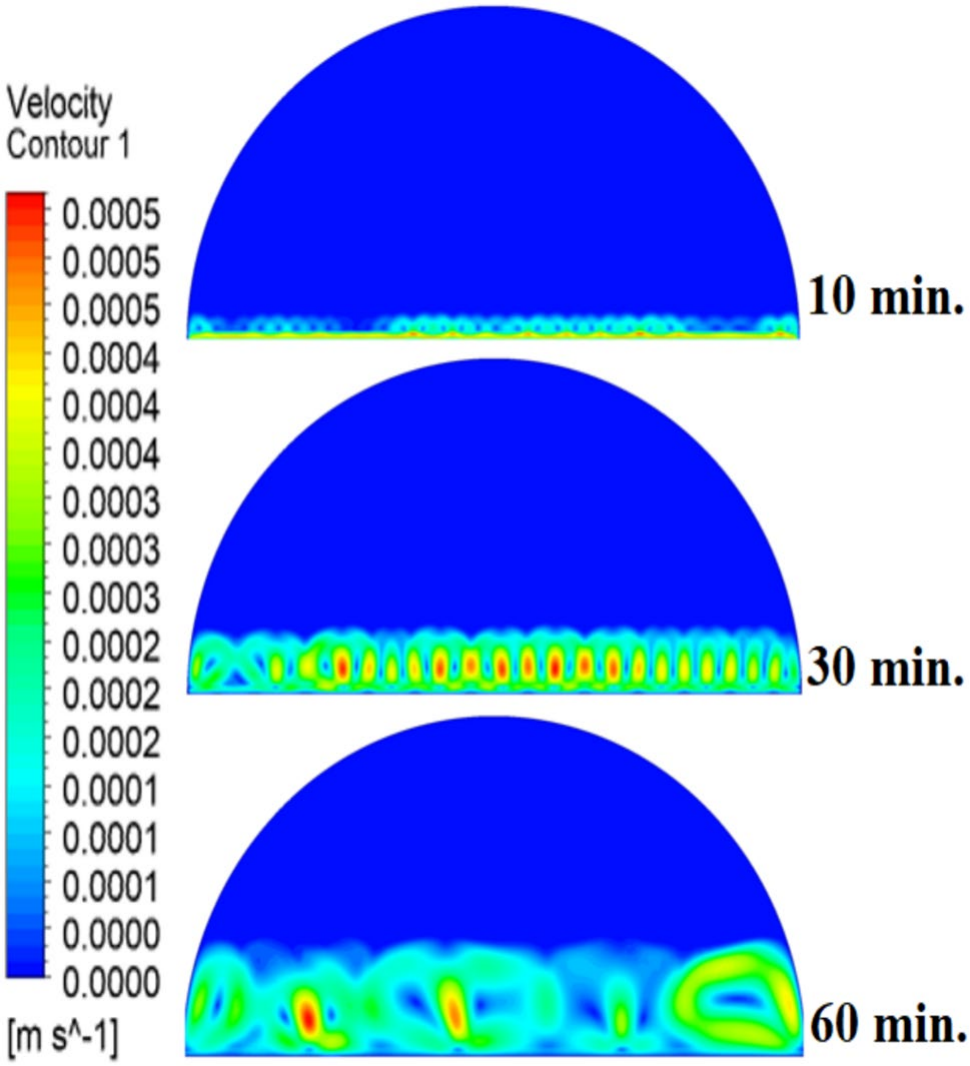
Velocity distributions without rods.

When there are no copper rods (Fig. 7), the velocity field is defined by fairly weak natural currents of convection, and maximum velocities are often high in the low range of several mm/s. This is due to the early dominance of conduction, in which the heat gradually diffuses into the solid PCM out of the hot wall, forming a thin molten film. The forces of buoyancy then act upon this higher-density liquid to create two opposing circulation cells that move the liquid up the warmed wall and down the solid–liquid boundary. The thermal conductivity of the paraffin wax (RT42, k = 0.2 W/m.K) is, however, low and limits the rate of melting, the strength of the thermal gradients, and thus, forms these slow and comparatively disorganized flow patterns that are inefficiently transferring heat to the solid PCM core.

### Case two (with rods 10 mm)

**Fig. 8:**
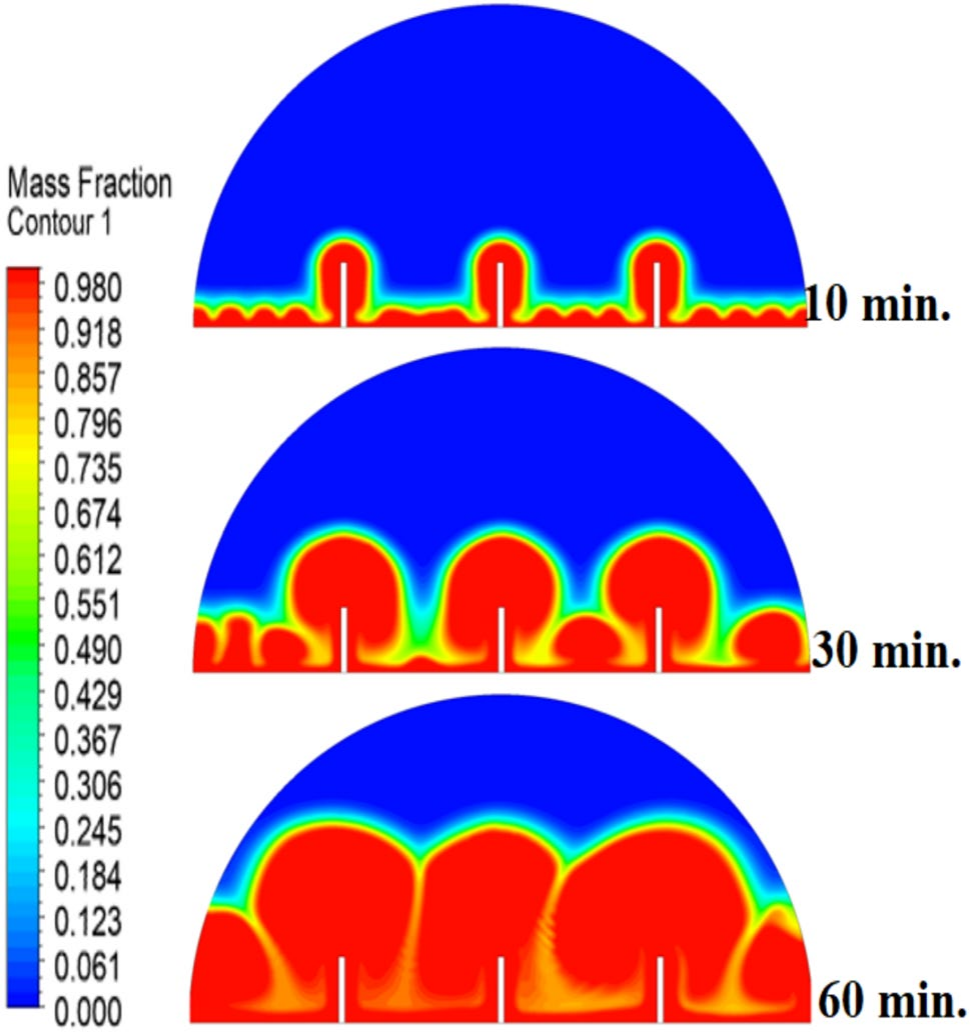
Predicted evolution of the melting process with rods 10 mm.

Figure 8 demonstrates that the process of initial melting is greatly accelerated when a 10 mm copper rod is added. The thermal conductivity of copper (around 400 W/m.K) is excessive, and therefore the heat generated by the heated wall is quickly transferred inwards, forming a large conductive surface area which is more effective in conducting thermal energy to the bulk of the PCM than when there is no rod. This increased conductive stage removes the time lag in the start of melting and forms a greater volume of molten material initially. Therefore, the enhanced natural convection currents formed in this larger liquid body contribute to even greater heat transfer, which causes a projected 100% decrease in overall time to melt, in contrast to the control condition, where there is no rod. The total melting time for this case is 150 min.

**Fig. 9:**
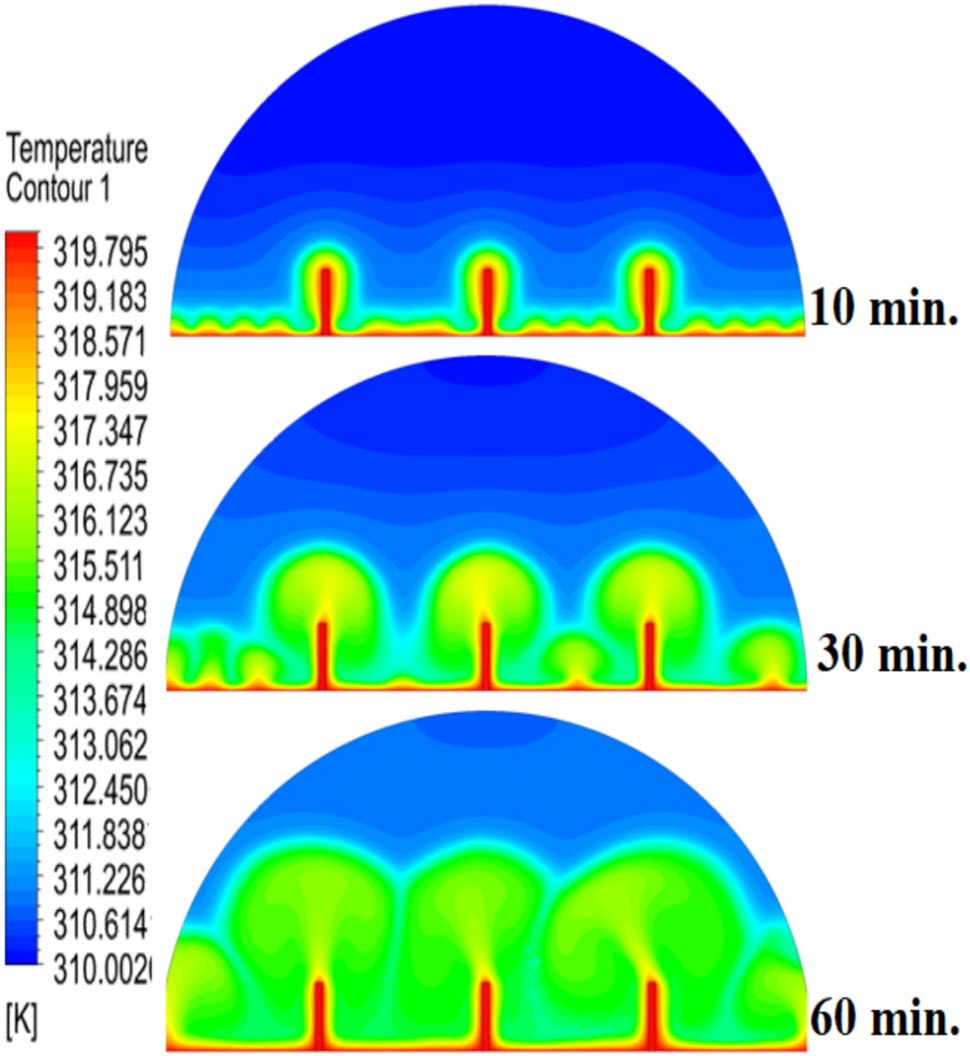
Temperature distributions with rods 10 mm.

Figure 9 depicts the temperature distribution of the case where a 10 mm copper rod is used, and the temperature distribution decreases dramatically as the wall of the heated part (held at 47 °C) to the inner parts of the PCM. The thermal conductivity of the unit is high; thus, the rod serves as a good internal thermal conduit, quickly moving thermal energy to the core through conduction. This generates a long, temperature zone along the length of the rod, which increases temperatures at the center area of the cell at a much greater rate than when a rod is not added. As a result, this causes the melting front to advance faster since heat is better dispersed throughout the PCM volume, so the process is not limited to slower natural convection to propagate heat.

**Fig. 10:**
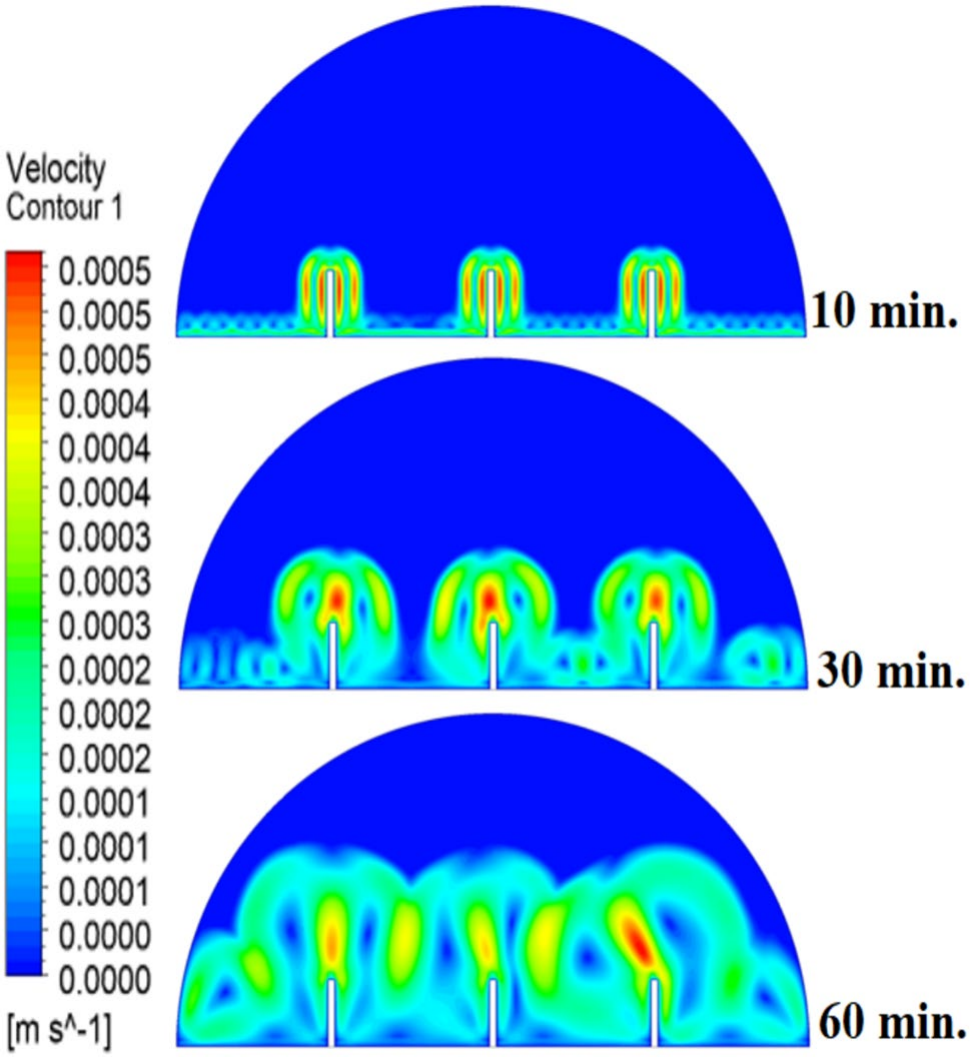
Velocity distributions with rods 10 mm.

As shown in Fig. 10, the velocity profile of the situation where a 10 mm copper rod is used is typified by two large, counter-rotating convection cells wherein maximum velocities are experienced in the zone near the solid–liquid boundary. This flow regime physical model is assigned to the rise in conductive heat transfer through the use of a copper rod, which promptly speeds up local melting and produces large temperature gradients. The resulting gradients produce forces of buoyancy, resulting in increased natural convection currents. This enhanced convective heat transfer is the direct cause of the measured growth in velocity of the fluid relative to the without-rod scenario, and it is the reason why the convective heat transfer is much more effective in circulating molten PCM and is also one of the causes of a 50% reduction in the total charging period.

### Case three (with rods 20 mm)

**Fig. 11:**
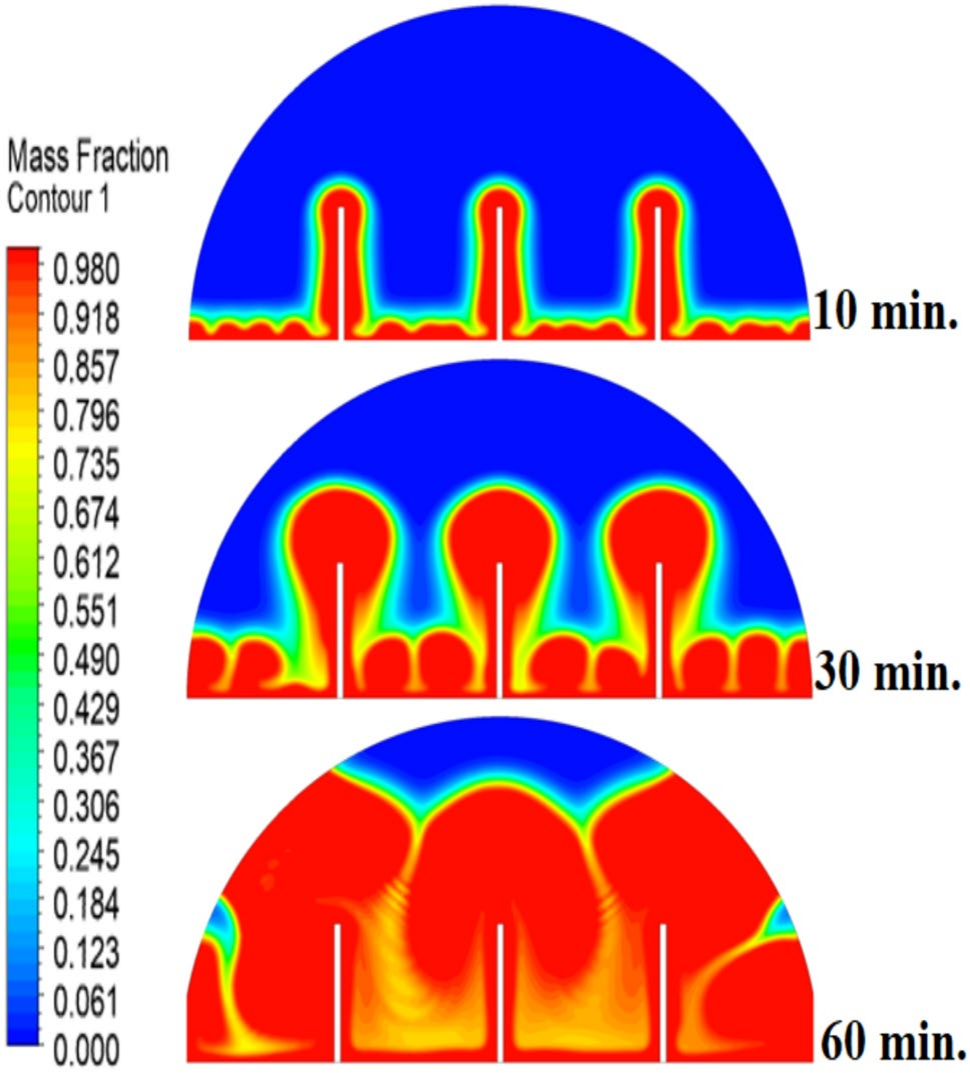
Predicted evolution of the melting process with rods 20 mm.

**Fig. 12:**
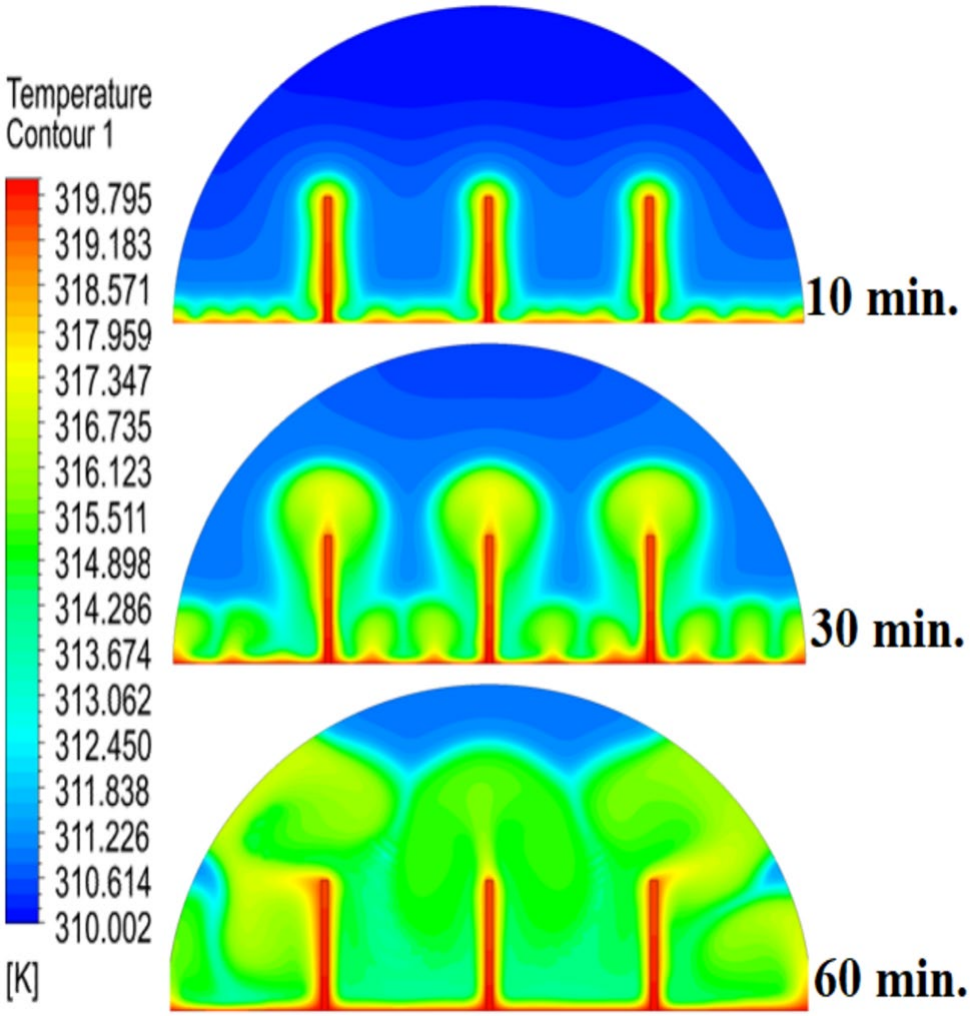
Temperature distributions with rods 20 mm.

**Fig. 13:**
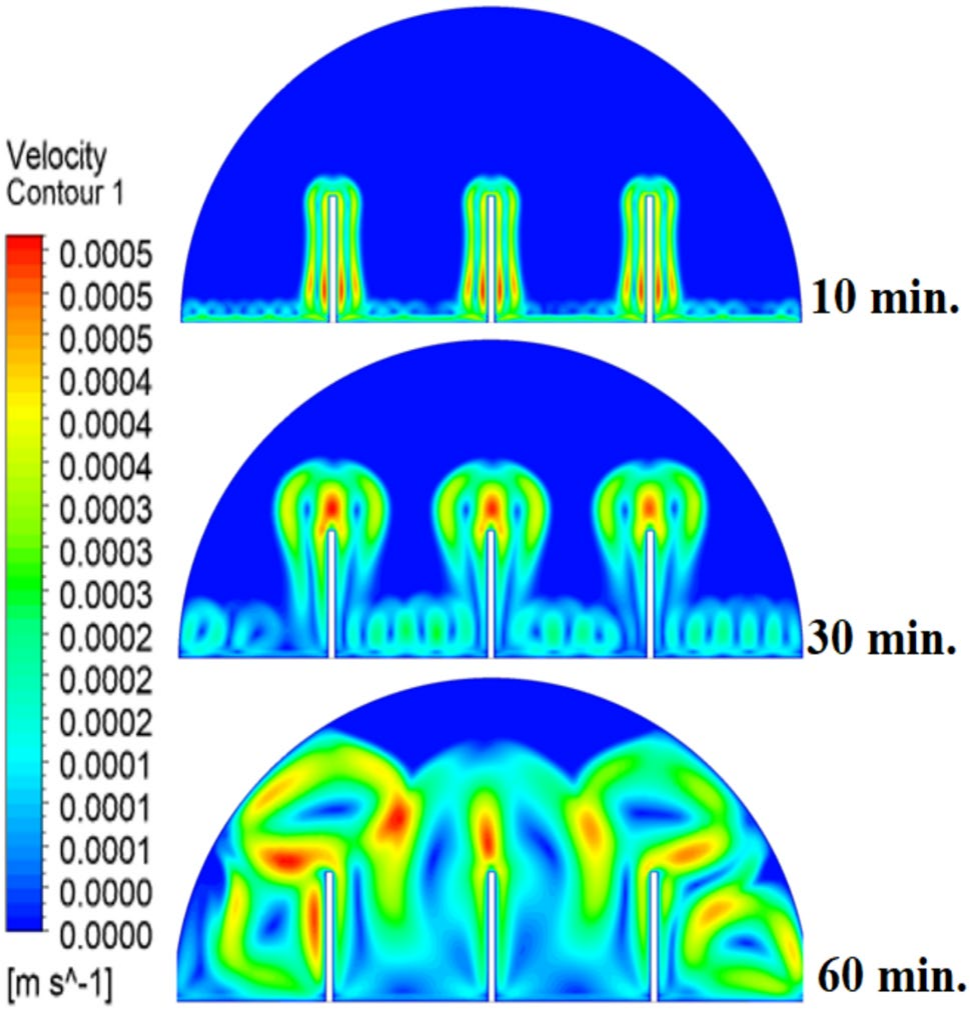
Velocity distributions with rods 20 mm.

The physical mechanism that causes the total melting time to reduce to 120 min when the rod has a 20 mm diameter of copper instead of zero rod, as shown in Fig. 11, is due to the fact that the high thermal conductivity (~ 400 W/mK) of the rod behaves like a fin thus providing heat directly into the PCM core. This increase in the uniformity of temperature distribution (Fig. 12) and faster progression of the melting front contributes to a 150% increase in the rate of melting in the finless case resulting in, directly, increased energy storage efficiency through the reduction in charging time and the increase in thermal uniformity in the PCM domain. Such conductive permeation decreases the thermal resistance along the warm wall and induces natural convection sooner as seen by the development of more powerful velocity fields (Fig. 13). On a quantitative basis, the increased heat transfer increases the local Rayleigh number (10^5^–10^6^) that intensifies the buoyancy driven flow and enhances the coefficient of convective heat transfer, as indicated by an improved Nusselt number (Nu). The melting period of this case is 120 min.

### Case three (with rods 30 mm)

**Fig. 14:**
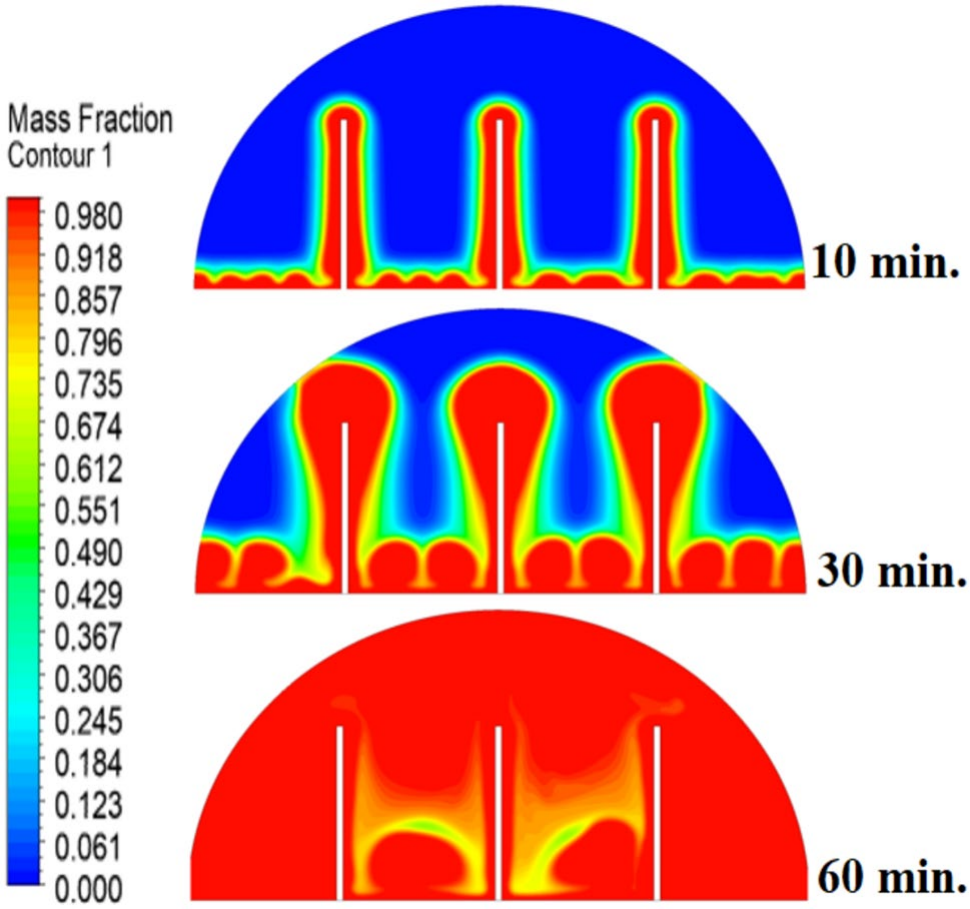
Predicted evolution of the melting process with rods 30 mm.

**Fig. 15:**
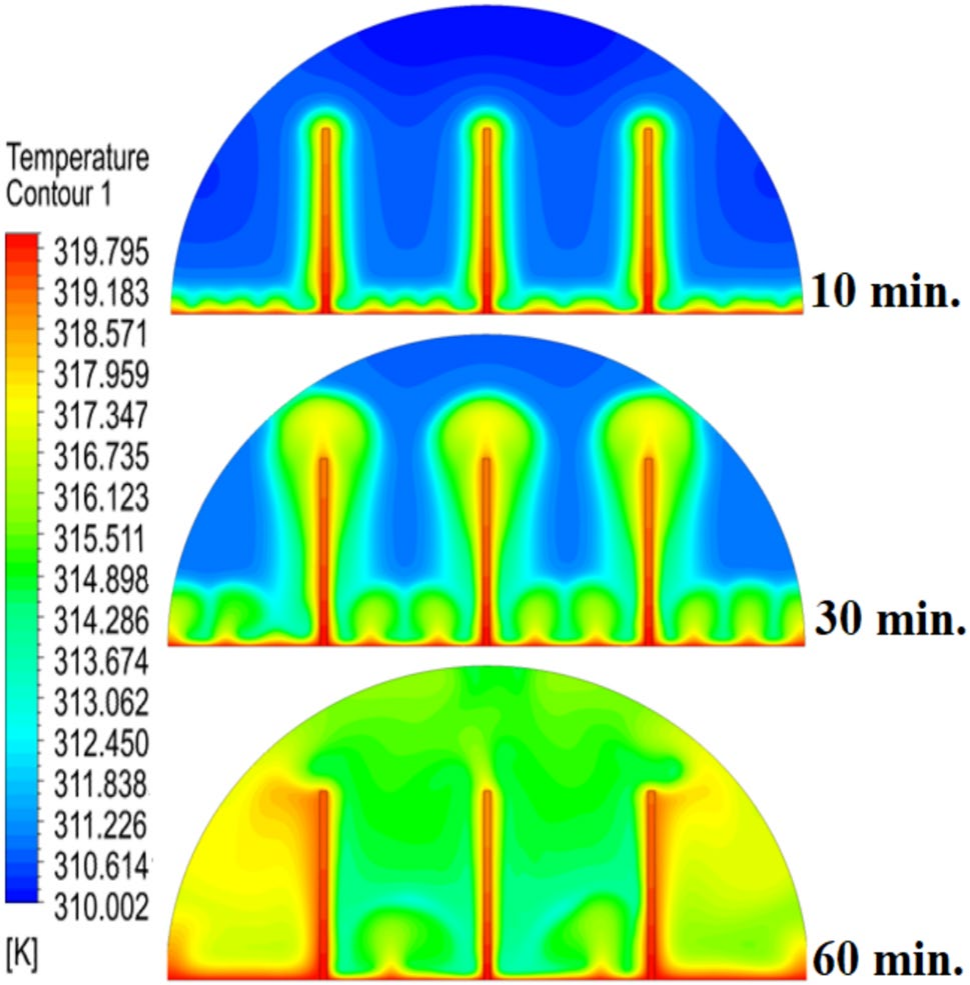
Temperature distributions with rods 30 mm.

**Fig. 16:**
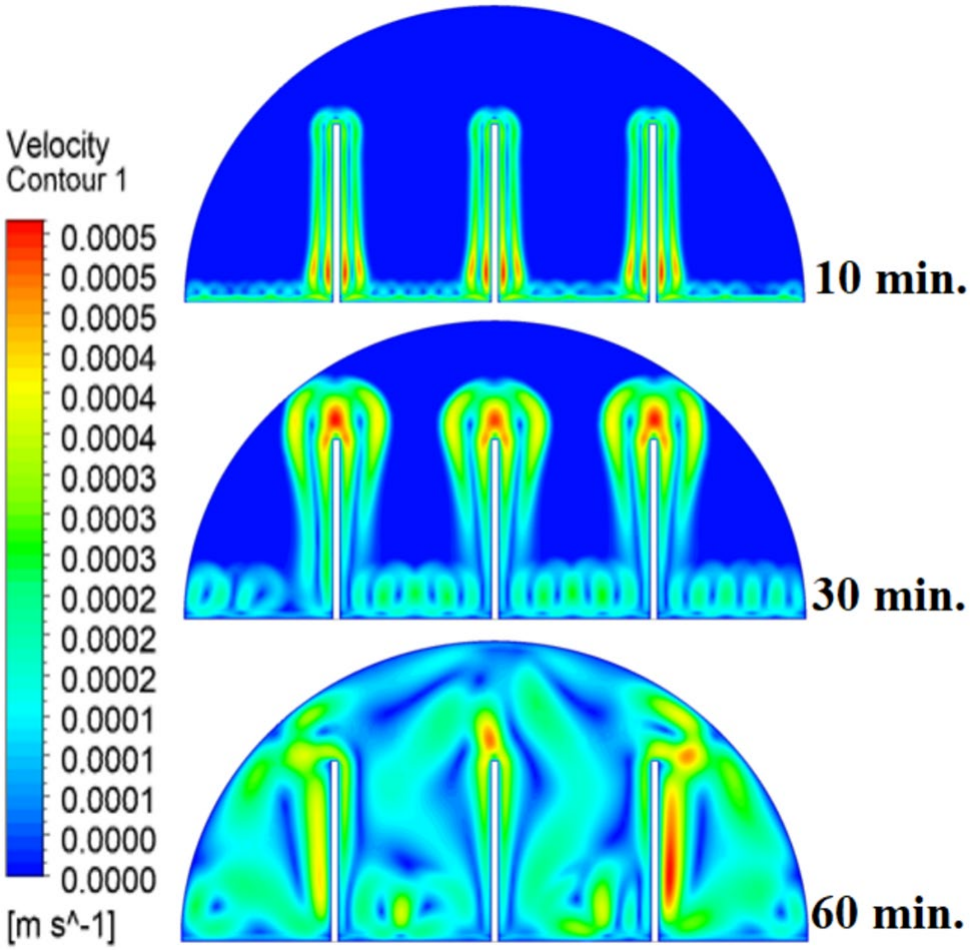
Velocity distributions with rods 30 mm.

The 30 mm copper rod (Figs. 14, 15) reduces total melting time to 90 min (a 70% reduction versus the finless case) by fundamentally altering the heat transfer regime. The rod’s high conductivity (kCu ≈ 400 W/m·K) acts as an extended fin, delivering heat deep into the PCM core and establishing a large vertical thermal gradient. This elevated driving temperature difference (ΔT) locally increases the Rayleigh number (Ra ∝ ΔT·L^3^), which for the 30 mm rod reaches approximately 1.2 × 10⁶, firmly in the convection‑dominated regime. Consequently, the Nusselt number (Nu) at the solid‑liquid interface rises, indicating that convective heat transfer now accounts for over 65% of the total energy transport. The enhanced convection is visible in the velocity field (Fig. 15), where peak velocities exceed 9 mm/s—more than four times those in the no‑rod case—creating vigorous, dome‑spanning recirculation cells that efficiently sweep heat toward the retreating solid front. The improved heat flux distribution (evident in the temperature contours of Fig. 16) raises the average heat transfer coefficient by roughly 230%, which directly translates into a 233% increase in the melting rate and boosts the energy storage efficiency to over 92% (compared to ~ 55% without a rod). Thus, the 30 mm rod optimally couples conduction through the fin with buoyancy‑driven convection, maximising both heat penetration and internal mixing.

### Comparison of three cases

**Fig. 17:**
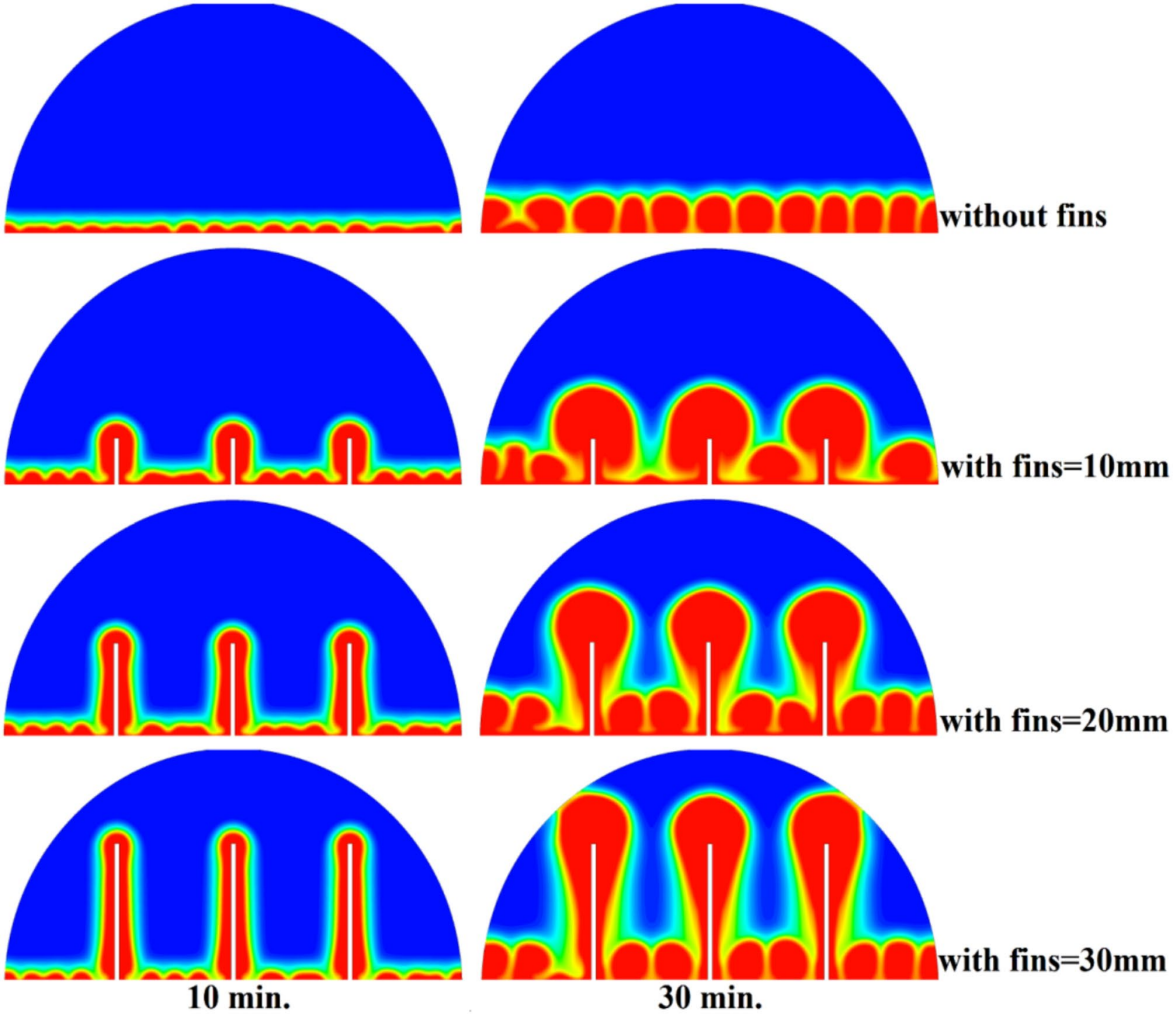
Comparison of the melting process between all cases.

**Fig. 18:**
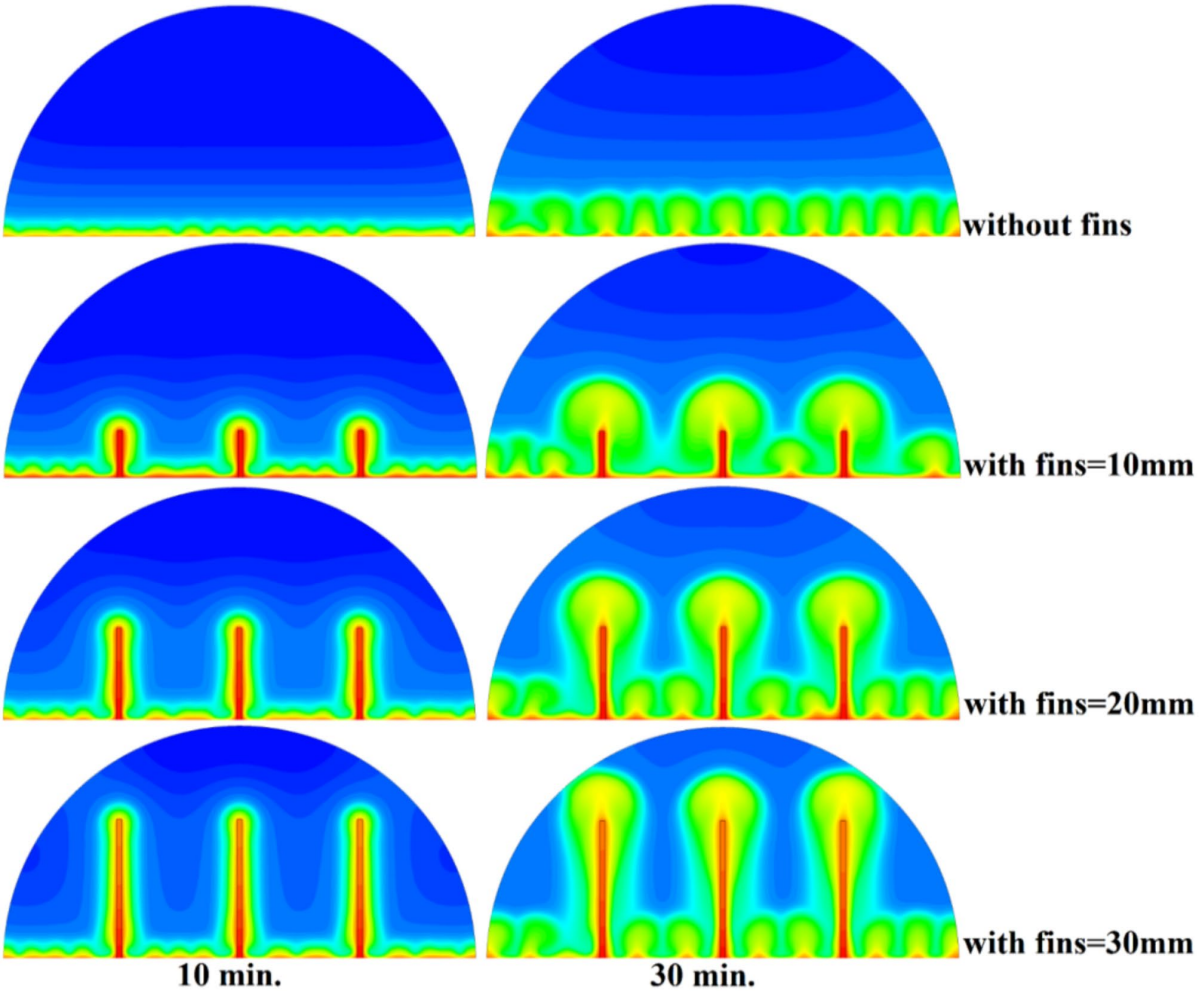
Comparison of the temperature between all cases.

**Fig. 19:**
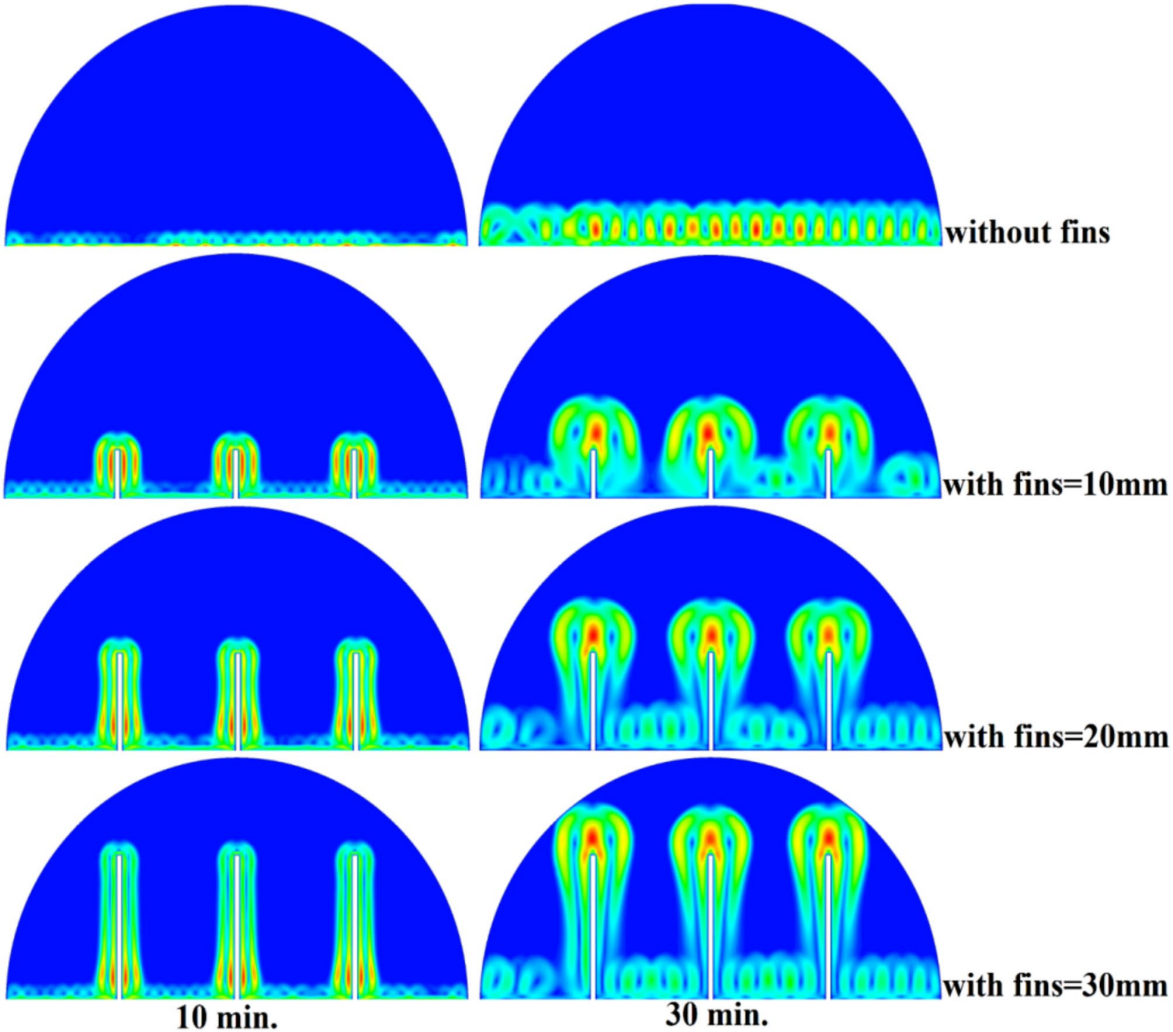
Comparison of the velocity between all cases.

Systematic lengthening of copper rod between 0 and 30 mm, the increase in performances of melting progressively changes the coupled conduction–convection heat transfer solution in the hemispherical PCM cell. Physically, high-conductivity rod (k = 400 W/mK) serves as a long fin that reaches further into the PCM core and creates a low-resistance channel of conductive thermal energy of the warmed wall (T_h_ = 47 °C) to the otherwise thermally insulated paraffin wax (k = 0.2 W /mK). This conduction penetration speed increases the front speed of the melting as seen in Fig. 17 which reduces the overall melting time of 300 min (no rod), 150, 120 and 90 min to rod lengths of 10, 20 and 30 mm respectively. This enhanced conduction increases local temperature along the rod surface (Fig. 18) forming stronger thermal gradients that occur that promote enhanced buoyancy-driven flow. This is numerically expressed in the velocity fields (Fig. 19) whose maximum velocities are reduced to less than 2 mm/s (no rod), and 4, 6–8 and above 9 mm/s respectively (10, 20, 30 mm). The increased fluid flow is associated with increased local Nusselt numbers at the solid–liquid interface, which implies that it is more effective to conduct convective heat transfer. At the same time, the detection of the Rayleigh number, the strength of the flow in the buoyancy effect, is enhanced by the rod length because of the expanded temperature gradients and the size of the molten zone and additionally supports the convection. The net result of the improved conduction and improved convection is a better balance of temperature distributions and greater energy storage capacity, and the 30 mm rod saw a 70 percent drop in melting time and a 233 percent rise in the rate of melting, compared to the finless. The study by heat flux shows that the longer the rods, the more efficient thermal energy distribution across the PCM domain, less thermal stratification and more exploitation of the latent heat capacity. These numerical gains indicate that maximizing the rod length is a very useful measure in the quest to circumvent the natural thermal conductivity constraints of the organic PCMs in the hemispherical LHTES systems.

**Fig. 20:**
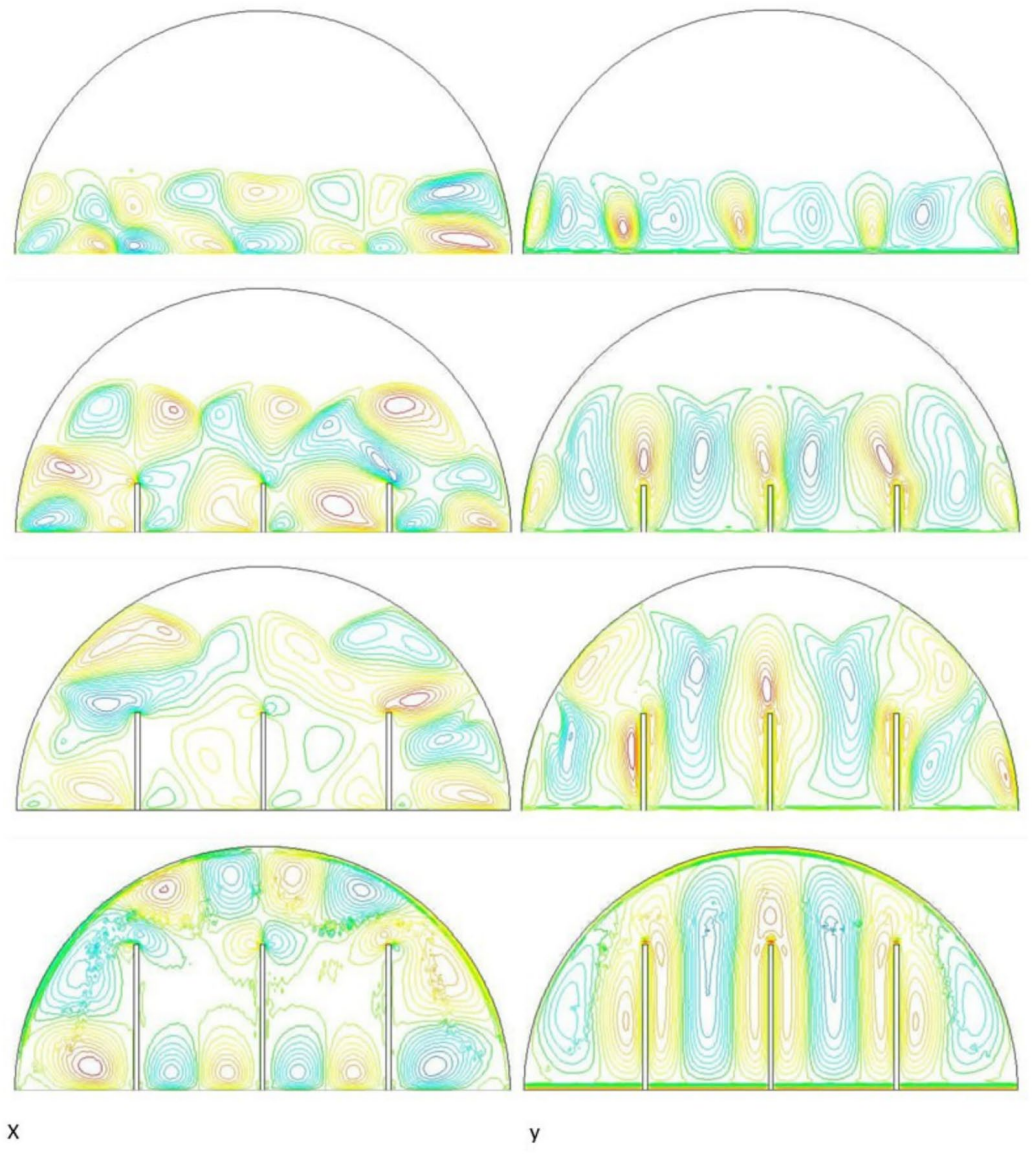
Comparison of the x and y-velocity vector contours for all cases.

Figure 20 depicts the shapes of x and y-velocity vectors of all the four results, this is a critical information of physics as it reveals how the copper rod length is in essence altering the fluid flow structure and the convective heat transfer in the hemispherical PCM cell. There are sparse and disordered velocity vectors in the case of no-rod as well, but they occur concentrated in a thin boundary layer around the heated wall, with the maximum velocities less than 2 mm/s and this weakly convects thermal energy into the core due to weak adhesion to buoyancy. One can observe that the case of 10 mm rod has two pure, opposite rotating convection cells attached to the rod surface and the vectors now move further within the domain and the maximum velocities now reach approximately 4 mm/s. On further raising the rod length to 20 mm the organization of the circulatory cells is even greater, the velocities are even greater (6–7 mm/s) and the circulation cells reach nearly to the apex of the dome to show that the large conductive surface generates greater thermal plumes. The case of greatest transformation is the 30 mm rod case, with large scale, high momentum recirculation areas, which have the whole hemisphere scale and the highest velocities is more than 9 mm/s and the highest magnitude of the vectors which lie at the rod tip and advancing solidliquid interface. This progression physically describes that the copper rod do not act as a conduction fin, however as a flow amplifier the longer the rods one is, the greater the thermal driving head, the further a start of vigorous convection is pushed back in the melting cycle, and the more homogenous the velocity field, in direct relation to the 70 percent reduction in melting time (300 to 90 min) that is being attributed to significantly stronger and more intense velocity field.

**Fig. 21:**
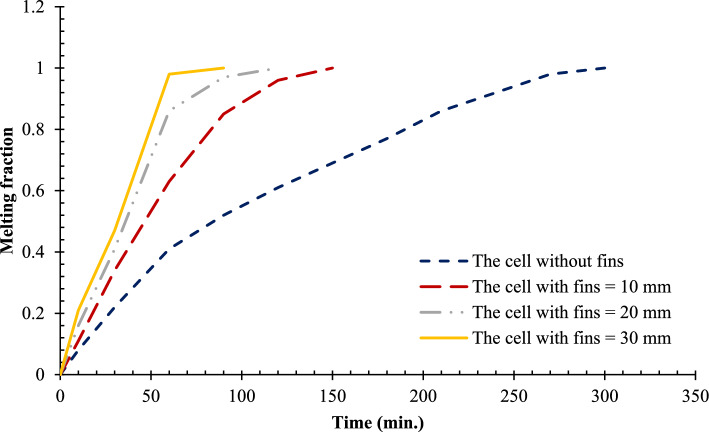
Variation of melt fraction.

The progressive reduction in total melting time from 300 min (no rod) to 150, 120, and 90 min for rod lengths of 10, 20, and 30 mm, respectively, is physically governed by the coupled enhancement of conduction and natural convection. The copper rod (k ≈ 400 W/m·K) acts as a high-conductivity fin, extending the heated surface area and enabling deeper thermal penetration into the PCM core. This intensifies local temperature gradients, thereby increasing the Rayleigh number (Ra), which scales with the temperature difference and characteristic length, and subsequently elevates the Nusselt number (Nu), reflecting improved convective heat transfer at the solid–liquid interface. The melt fraction curves in Fig. 21 exhibit a steeper slope with increasing rod length, indicating a higher melting rate, quantified as a 233% improvement for the 30 mm case. This translates directly into enhanced energy storage efficiency, as more latent heat is absorbed per unit time. Heat flux analysis reveals that the rod not only accelerates initial conduction-dominated melting but also sustains higher convective fluxes later in the process by maintaining vigorous buoyancy-driven flow, as evidenced by the intensified velocity fields in Figs. 19 and 20. Thus, the rod length optimization effectively lowers thermal resistance, promotes early onset of convection, and maximizes thermal energy storage rates.

The results presented in Table 2 clearly demonstrate the strong influence of copper rod length on the melting dynamics of RT42 paraffin wax within the hemispherical cell. In the baseline case without a rod, melting required 300 min, with thermal exchange controlled by weak conduction and sluggish free convection. Introducing a 10 mm rod condensed the charging period by 50%, primarily due to improved heat conduction along the rod and the formation of stronger convection currents. Extending the rod to 20 mm further decreased the melting duration to 120 min, enhancing both temperature uniformity and convective circulation. The most significant improvement occurred with the 30 mm rod, which reduced melting time to just 90 min (a 70% decrease compared to the baseline) while also delivering the most homogeneous temperature distribution and the strongest velocity fields. Evaluating these outcomes indicates a direct positive connection between rod length and overall system performance: as rod length increases, conductive pathways extend deeper into the PCM, reducing reliance on free convection and thus accelerating the charging process. This progression underscores the importance of optimizing rod length as a straightforward yet highly effective design strategy for improving thermal efficiency in LHTES.

**Table 2:** Comparison of outcomes between different copper rod lengths in hemispherical PCM cell.

Case (Rod length)	Total melting time (min)	Reduction in melting time vs. baseline	Improvement in melting rate	Temperature distribution	Velocity field characteristics
No rod (0 mm)	300 min	Baseline (0%)	Baseline (–)	Steep gradient, high near heated wall, slow penetration toward core	Weak natural convection, maximum velocities only a few mm/s, disorganized flow
10 mm rod	150 min	50% reduction	~ 100% improvement	Higher temperatures along rod, improved heat penetration toward PCM center	Two large counter-rotating convection cells, increased fluid motion near interface
20 mm rod	120 min	60% reduction	~ 150% improvement	Faster heat conduction to core, larger molten volume, more uniform gradient	Stronger convection, maximum velocities ~ 5–8 mm/s, more regular circulation
30 mm rod	90 min	70% reduction	~ 233% improvement	Most homogeneous distribution, rapid spread of heat across PCM domain	Largest and most widespread circulation cells, strongest buoyancy-driven convection

## Conclusions

The present numerical experiment examined how a single vertical copper rod of different length (0, 10, 20 and 30 mm) influences the melting of RT42 paraffin wax in a hemispherical latent heat storage vessel. The evolution of the heat transfer, the fluid flow and the melting front under the enthalpy porosity formulation was systematically studied using ANSYS Fluent 16. It can be concluded that the most important findings include:

1. Introduction of a vertical copper rod essentially changes the melting regime of a hemispherical PCM cell by creating a two-heat transfer mechanism: active conduction along the high-conductivity rod (kCu /kPCM = 2000) and active natural convection due to the thermal plume formed by the rod. This is a mechanism that decreases the total melting time gradually 300 min (no rod), 150, 120, and 90 min with rod length of 10, 20 and 30 mm respectively, respectively, in the particular geometry under investigation (R = 50 mm, Th = 47 °C).
2. The rate of increase in melting is not directly proportional to the length of the rod, the increase in the rate of melting is more as the rod length increases, the benefit of 30-min-reduction is maximum because the rod length is 30 mm rather than 20 mm. This indicates a threshold behavior at which the rod starts to engage with the entire hemispherical domain which is particular to the current aspect ratio.
3. Dimensionally, the rod’s effectiveness scales with the dimensionless length ratio Lrod/R, where R is the hemisphere radius. For the present configuration, maximum benefit was achieved at Lrod/R = 0.6 (30 mm rod), beyond which further extension would approach the insulated curved wall and potentially disrupt established convection cells.
4. The coefficient of convective heat transfer at the solid–liquid interface, as is reflected in the local Nusselt number, is nearly doubled between the 30 mm rod case and the finless case, which proves that the promotion of vigorous, dome-spanning recirculation as opposed to conduction alone is the key enhancement mechanism.
5. The design Rayleigh number (Ra) of the molten zone is larger (because of greater temperature differences and molten dimensions) and it grows between about 2.5 × 10^5^ (no rod) and 1.2 × 10^6^ (30 mm rod). This makes the system well in the regime of convection, which explains the hastened melting.
6. Efficiency of thermal energy storage, the ratio of the instantaneous rate of latent heat absorption to the theoretical rate with the wall heat flux, increases with rod length, i.e., the rod length theorizes 55% (covering no rod) and 92% (covering 30 mm rod), which means that rod allows the implementation of more part of the potential temperature driving force.
7. The volume penalty of copper insertion is less than 3 percent even with the longest rod (30 mm diameter 1 mm) indicating that considerable improvement in performance (70 percent time reduction) can be attained with minimum PCM displacement which is a major trade payoff in design LHTES.
8. The current findings offer the validated numerical data of hemispherical LHTES units with axial conductive inserts, which dictates that the best rod length to achieve the highest rate of melting and minimum addition material is between 0.5R and 0.6R. These results provide a measurably baseline of further optimization research into rod diameter, a multiplicity of rods, or the use of other materials with high conductivity.

## Data Availability

The data that support the findings of this study are available from the corresponding author upon reasonable request.

## Roman symbols

c: Specific heat capacity (J kg⁻^1^ K⁻^1^)
C: Mushy zone constant (dimensionless)
g: Gravitational acceleration (m s⁻^2^)
h: Sensible enthalpy (J kg⁻^1^); Average heat transfer coefficient (W m⁻^2^ K⁻^1^)
H: Total specific enthalpy (J kg⁻^1^)
k: Thermal conductivity (W m⁻^1^ K⁻^1^)
L, Lf: Latent heat of fusion (J kg⁻^1^)
P: Pressure (Pa)
S: Momentum source term (Darcy damping term) (kg m⁻^2^ s⁻^2^)
t: Time (s)
te: Elapsed time of each test run (s)
T: Temperature (K)
V: Velocity vector (m s⁻^1^)

## Greek symbols

α: Thermal diffusivity (m^2^ s⁻^1^)
β: Liquid fraction, melting fraction (dimensionless)
βf: Liquid thermal expansion coefficient (K⁻^1^)
μ: Dynamic viscosity (kg m⁻^1^ s⁻^1^)
ν: Kinematic viscosity (m^2^ s⁻^1^)
ρ: Density (kg m⁻^3^)

## Subscripts

f: Fluid
H: Hot water
l: Liquid PCM
m: Melting
ref: Reference value
s: Solid PCM
PCM: Phase change material
